# Positioning of psychodynamic psychotherapy in the treatment of depression: A comparison of the RANZCP 2020 and NICE 2022 guidelines

**DOI:** 10.1177/10398562231159329

**Published:** 2023-02-24

**Authors:** Gin S Malhi, Erica Bell, Darryl Bassett, Philip Boyce, Philip Hazell, Malcolm Hopwood, Bill Lyndon, Roger Mulder, Richard Porter, Greg Murray

**Affiliations:** Academic Department of Psychiatry, Kolling Institute, Northern Clinical School, Faculty of Medicine and Health, 4334 The University of Sydney, St Leonards, NSW, Australia; CADE Clinic and Mood-T, Royal North Shore Hospital, St Leonards, NSW, Australia; Department of Psychiatry, 6396 University of Oxford, Oxford, UK; Academic Department of Psychiatry, Kolling Institute, Northern Clinical School, Faculty of Medicine and Health, 4334 The University of Sydney, St Leonards, NSW, Australia; CADE Clinic and Mood-T, Royal North Shore Hospital, St Leonards, NSW, Australia; Faculty of Health and Medical Sciences, 532966 University of Western Australia, Perth, WA, Australia; Discipline of Psychiatry, Sydney Medical School, Faculty of Medicine and Health, 4334 University of Sydney, Sydney, NSW, Australia; Discipline of Psychiatry, Sydney Medical School, Faculty of Medicine and Health, 4334 University of Sydney, Sydney, NSW, Australia; Department of Psychiatry, University of Melbourne and Professorial Psychiatry Unit, Melbourne, VIC, Australia; Discipline of Psychiatry, Sydney Medical School, Faculty of Medicine and Health, 522555 University of Sydney, Sydney, NSW, Australia; Department of Psychological Medicine, 2494 University of Otago, Christchurch, New Zealand; Department of Psychological Medicine, 2494 University of Otago, Christchurch, New Zealand; Centre for Mental Health, 3783 Swinburne University of Technology, Hawthorn, VIC, Australia

**Keywords:** Guidelines, depression, psychodynamic psychotherapy, treatments

## Abstract

**Objective:**

To compare the 2022 NICE guidelines (NG222) and 2020 RANZCP clinical practice guidelines (MDcpg^2020^) recommendations for the treatment of depression using psychodynamic psychotherapy.

**Conclusions:**

Both guidelines recommend psychological interventions first-line. However, only short-term psychodynamic psychotherapy (STPP) is recommended, and in the NG222 it is ranked last for less severe depression and 7th for more severe depression. In contrast, cognitive behavioural therapy and behavioural activation are deemed the more clinically effective and cost-effective psychological therapies. And antidepressants play a significant role – largely in more severe depression.

Psychodynamic psychotherapy is a longstanding psychological intervention within psychiatry. As such, its positioning in the treatment of depression is important. However, whether it should be recommended within treatment guidelines and what form of therapy (short or long term) is supported by evidence, has been a point of contention with prior discussion in the pages of Australasian Psychiatry. Therefore, to provide an independent perspective, the recommendations made by the College guideline for mood disorders (MDcpg^2020^) are compared to those made by the recently published NICE guidelines.^1^ The latter were chosen because of the rigour with which the evidence was examined and because the time period surveyed overlapped with that examined by the MDcpg^2020^.

*NICE guidelines (UK)*: On June 29^th^ 2022, the National Institute for Health and Care Excellence (NICE) published guidelines for the management of depression (NG222).^2^ These guidelines were developed with extensive input from a broad range of stakeholders. The culmination of this tremendous effort is a document that provides succinct, clear guidance for the management of depression that is based on a detailed analysis of pertinent evidence.

## Positioning of STPP

This brief article focuses on the positioning of STPP in the management of acute depression. First, it compares the *approach* taken by the MDcpg^2020^ and the NG222 with respect to the management of depression using psychological interventions. Second, the *recommendations* made by the NG222 are compared to those in the MDcpg^2020^. Third, and finally, some key points are provided – noting that by and large the NG222 reaffirm the MDcpg^2020^ recommendations.

### Comparison of the approach taken by the MDcpg^2020^ and the NG222

#### Diagnosis

As would be expected, the NG222 underscore the importance of careful evaluation and accurate diagnosis. And as regards management, they follow the MDcpg^2020^ lead by assigning primacy to psychological treatments where available and emphasise the importance of a broader therapeutic approach that includes lifestyle interventions such as diet and exercise. Further, in a similar fashion to the MDcpg^2020^, the NG222 aim to inform and advise. In other words, they are not prescriptive per se, although clearly whenever possible, it is common sense to pursue strategies where there is evidentiary support.

In the NICE guidelines depression is defined broadly according to the criteria stipulated in the International Classification of Diseases 11 (ICD-11)^3^ and the Diagnostic and Statistical Manual of mental disorders (DSM-5).^4^ Depression is taken to refer to *‘a wide range of mental health problems characterised by the absence of a positive affect (a loss of interest and enjoyment in ordinary things and experiences), low mood and a range of associated emotional, cognitive, physical, and behavioural symptoms’*.^2^ In addition to this syndromic definition, *severity* is mapped on a continuum that consists of three elements: these are the *symptoms* which can vary in frequency and intensity, the *duration* of the depressive disorder, and the *impact* it has on personal and social functioning. In other words, severity is not simply a function of the number of symptoms but also captures chronicity and the extent of impairment that the illness confers.

This is very similar to the MDcpg^2020^ in which depression is diagnosed according to a number of schema that are complementary and include DSM-5, severity, and subtypes that are defined according to clinical profiles. In addition, the MDcpg^2020^ allowed for the ACE model that emphasises similar domains to ICD-11 and provides a further dimensional aspect to severity and syndromic classification.

Consequently, the NG222 partitions new episodes of depression as ‘*less severe* or *more severe* depression’. These categories are groupings of previously used descriptors – sub-threshold, mild, moderate and severe. In this new grouping, less severe refers to sub-threshold and mild depression, whereas more severe refers to moderate and severe depression. The reason for dividing depression as such, is that the guideline developers wanted to reflect the evidence pertaining to classification and further, use a division that would enhance the uptake of guideline recommendations in clinical practice.

Again, this approach is similar to the stance adopted by the MDcpg^2020^, in which subtypes are acknowledged but the heterogeneity of the illness is respected and it is recognised that other than very specific kinds of depression such as psychosis and melancholia, in the main, depression is best treated more broadly and largely according to the functional impairment it confers. Thus, a single overarching schema is presented for the management of acute depression that systematically builds through Actions, Choices and Alternatives. In this schema, the clinician decides on the basis of information available to them how to characterise the illness and formulate its management.

#### Efficacy, effectiveness and cost

A key difference, and an important additional component that NG222 provides as compared to MDcpg^2020^, is the incorporation of cost alongside effectiveness. When evaluating treatments and management strategies, the MDcpg^2020^ focused primarily on efficacy and side-effects (tolerability). These clinical considerations were given importance because in practice the first question most clinicians will (or at least should) consider is whether a particular treatment works. In other words, does it have proven efficacy. If so, then the second consideration is whether the patient can withstand suitable therapy for a sufficient period of time for it to have an effect. The combined consideration of efficacy and tolerability provides an indication of *effectiveness*. However, NG222 goes one step further and additionally factors the cost of the therapy. This is an increasingly important consideration and one that is perhaps especially relevant to the Australian landscape. Notably, the inclusion of cost provides a much clearer separation of the various therapies and strategies.

### The 2020 mood disorders CPG guidelines (MDcpg^2020^)

In the MDcpg^2020^, psychological interventions are a key component of Actions mandated in the management of depression. A key strength of *“the psychological component is that it offers an opportunity for learning new cognitive and behavioural skills to prevent future relapse and recurrence"* (section 7.1 page 62). Hence the MDcpg^2020^ identifies 6 psychological interventions (CBT, IPT, problem solving therapy, behavioural activation therapy, non-directive supportive therapy and STPP), chosen because each has been found to be more effective than wait list control in at least 10 RCT’s. The MDcpg^2020^ also note that in the outpatient treatment of major depression, there are no significant differences in the benefits derived from antidepressant therapy in comparison to psychological therapy.^5^ These recommendations within the MDcpg^2020^ therefore tally with those of the NG222.

## NG222 recommendations for the first line treatment of *less severe* depression in adults

**Figure 1.**
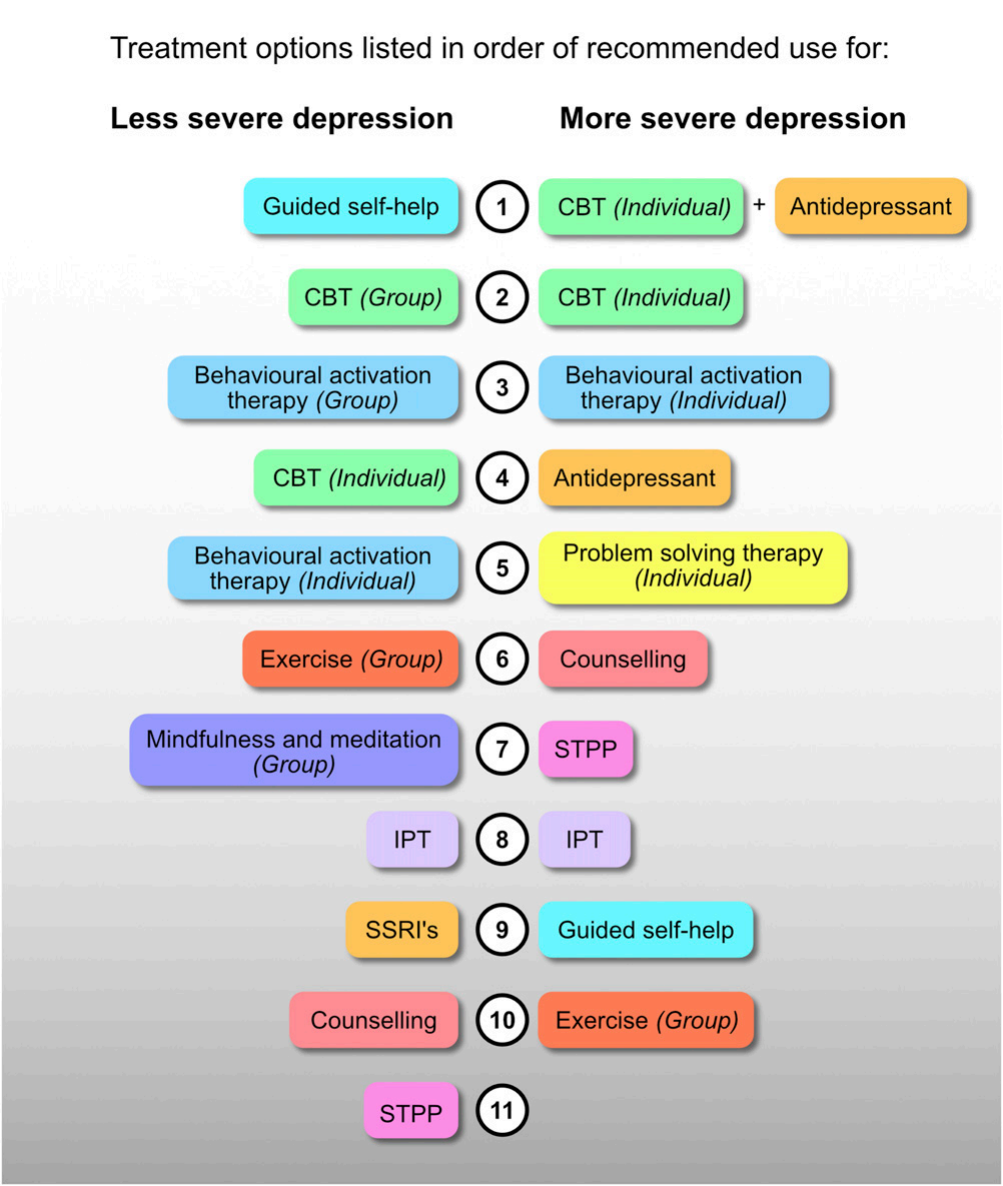
Schematic illustrating treatments ordered by recommended use according to NG222. Treatments are identified by separate colours, without distinguishing between mode of delivery (i.e. group or individual). In addition, SSRIs and the broader category of antidepressants are shown in the same colours for ease of interpretation. CBT = cognitive behavioural therapy; IPT = Interpersonal therapy; SSRI’s = selective serotonin reuptake inhibitors.

The NG222 provide a list of treatment options that are sequenced according to clinical and cost-effectiveness alongside consideration of implementation factors. The rank order of these various treatments is shown in Figure 1. In essence, group therapies are preferable, presumably largely because of cost savings. However, it is interesting to note that out of 11 first-line treatments for less severe depression, STPP is ranked last. Further, and importantly, SSRIs are positioned ahead of STPP and both individual and group CBT and behavioural activation therapy feature in the top four treatments recommended for the first-line treatment of less severe depression.

## NG222 recommendations for the first-line treatment of *more severe* depression in adults

The NG222 provides 10 first-line interventions. Broadly speaking group interventions do not feature other than group exercise, which is ranked as the least cost-effective option. This may be because more severe depression often requires tailored and in-depth therapy and patients are more likely to need hospitalization. Therefore, greater emphasis is placed on individual therapies. Antidepressant medication options also become broader when treating more severe depression, extending beyond SSRIs. Further, individual problem solving that was included in the MDcpg^2020^ is now included as a first-line treatment.

Relevant to our present discussion, STPP features seventh out of the 10 treatments. Further, antidepressant medication is now first line alongside individual cognitive behavioural therapy, which takes pole position both in combination with antidepressants and as individual therapy.

## Key points

1. Psychological interventions:
  1.1. Warrant priority and should be delivered first-line either individually or in groups depending on availability and need.
  1.2. Treatment should be personalised and tailored to individual needs.
2. Cognitive behavioural therapy and behavioural activation:
  2.1. These are the most clinically effective and cost-effective psychological therapies.
3. Psychodynamic Psychotherapy:
  3.1. Only STPP has a role in the treatment of acute depression.
  3.2. STPP ranks last for less severe depression and 7th out of 10 potential therapies for more severe depression.
  3.3. Counselling is ranked higher than STPP across the whole spectrum of depression (both less and more severe depression).
  3.4. Long term psychodynamic therapy is not indicated for acute depression irrespective of severity.
4. Antidepressants:
  4.1. Have a significant role to play in the management of depression, especially in more severe depression.
  4.2. When prescribed, antidepressants should be accompanied by psychological interventions where possible.

## Conclusion

Naturally, because of slightly different classificatory models there are subtle differences between the recommendations made by NG222 and by the MDcpg^2020^, but broadly the same therapies have been recommended and the importance attached to them is also consistent.
